# Renin-angiotensin-aldosterone system genes and nonarteritic anterior ischemic optic neuropathy

**Published:** 2011-05-06

**Authors:** Sofia Markoula, Sotirios Giannopoulos, Ioannis Asproudis, Charilaos Kostoulas, Alexios Nikas, Eleni Bagli, Athanassios P. Kyritsis, Ioannis Georgiou

**Affiliations:** 1 Department of Neurology, University of Ioannina School of Medicine, Ioannina, Greece; 2 Department of Ophthalmology, University of Ioannina School of Medicine, Ioannina, Greece; 3 Laboratory of Medical Genetics, University Hospital of Ioannina, Ioannina, Greece

## Abstract

**Purpose:**

Recent literature suggests a genetic component for non-arteritic anterior ischemic optic neuropathy (NAION). We examined the association of the insertion/deletion (I/D) polymorphism of the angiotensin-converting enzyme gene, of the M235T polymorphism of the angiotensinogen gene, and of the A1166C polymorphism of the angiotensin II type 1 receptor gene with NAION.

**Methods:**

Forty-seven patients with NAION and 76 controls, age- and gender-matched, were recruited and genotyped for renin-angiotensin-aldosterone system (RAAS) genes. Genotypes were determined by polymerase chain reaction and restriction enzyme analysis. NAION and control groups were compared in regard to the prevalence of renin-angiotensin-aldosterone system polymorphisms, and further stratified by age and gender.

**Results:**

NAION occurrence was not associated with the M235T polymorphism of the angiotensinogen gene and the A1166C polymorphism of the angiotensin II, type 1 receptor gene. Regarding the angiotensin-converting enzyme insertion/deletion polymorphism, our findings suggest that the II genotype could be a risk factor for NAION in younger male patients when compared to all cases and controls (p=0.033, odds ratio=5.71, confidence interval=1.152¨C28.35 and p=0.03, odds ratio=5.33, confidence interval=1.17¨C24.31 respectively). Furthermore I allele was present in all male patients younger than 55 years, making this allele a likely predisposing factor for NAION in young males.

**Conclusions:**

Since NAION may occur when compromised watershed microcirculation is combined with insufficient autoregulation of systematic circulation, polymorphisms of genes involved in systematic circulation, such as the RAAS genes, may be associated with NAION occurrence. Large-scale, multicentered, controlled prospective studies are needed to further explore the effects of *RAAS* polymorphisms or other genetic factors on NAION susceptibility.

## Introduction

Anterior ischemic optic neuropathy (AION), is a vision loss-threatening disease caused by an infarction of the optic nerve, primarily affecting patients older than 55 years [1]. Over a few days, optic disc edema develops, sometimes associated with flame hemorrhages of the swollen disc or nearby cotton-wool exudates. Visual loss is usually permanent, with minor recovery within the first weeks or months. Optic disc edema is followed within the next few weeks by optic atrophy of varying degrees that is generalized or sectorial [2].

Anterior ischemic optic neuropathy (AION) occurs in two forms: arteritic and nonarteritic (NAION). The arteritic type is associated with giant-cell arteritis, whereas in NAION cases, hypertension, diabetes mellitus, ischemic heart disease, dyslipidemia, hypercoagulable states, and sleep apnea syndrome have been described as risk factors [2-6]. Reports of the familiar NAION [7], with its predominance among whites [8] and its possible association with human leukocyte antigen (HLA)-A-29 [9], suggest a genetic component in NAION development. Therefore, NAION may occur when a genetic predisposition is combined with a variety of risk factors and anatomic variants, such as crowded optic disc syndrome [10].

Recent studies have examined, with conflicting results, the association of NAION with thrombophilic genes such as the methylenetetrahydrofolate reductase (*MTHFR*) C677T polymorphism and platelet polymorphisms on the glycoprotein Ib alpha gene [2,4,5,11], and with hypertension genes [12], hereditary deficiencies of protein C, protein S and antithrombin III, hyperhomocysteinemia, and primary antiphospholipid syndrome [5,6,13,14].

The renin-angiotensin system genes exhibit three common polymorphisms: the insertion/deletion (I/D) polymorphism of the angiotensin-converting enzyme (*ACE*) gene, the M235T polymorphism of the angiotensinogen gene (*AGT*), and the A1166C polymorphism of the angiotensin II type 1 receptor gene (*AT1-receptor*). There is sound evidence to indicate that variations of renin-angiotensin-aldosterone system (*RAAS*) genes may confer an increased risk for hypertension, preeclampsia, cardiovascular disease, and stroke [15-18].

In the present study, we examined the association of these three *RAAS* gene polymorphisms with NAION in a reference center in Northwest Greece, a well defined area with a homogeneous population and limited recent immigration.

## Methods

### Study participants

The NAION patients studied were recruited from January 2004 to December 2007. A detailed medical history was obtained and thorough physical examinations of all patients were performed. For diagnosis of NAION, all patients met the established criteria, including sudden visual loss, relative afferent papillary defect, visual-field defects consistent with ischemic optic neuropathy, and characteristic fundus changes (swollen, pale optic disc surrounded by splinter-shaped hemorrhages, followed by optic atrophy).

Exclusion criteria were erythrocyte sedimentation rates over 33 mm in the first hour for men and over 35 mm in the first hour for women, and any clinical or paraclinical evidence of autoimmune or rheumatic disease.

The epidemiological, clinical laboratory features and known risk factors for atherosclerosis (hypertension, diabetes mellitus, dyslipidemia) were evaluated in all patients with NAION. Arterial hypertension was defined when clearly documented (>140/90 mmHg) or treated. Diabetes mellitus was defined as present, if fasting glucose was greater than 126 mg/dl or was being treated with antidiabetic medication. Dyslipidemia was defined as fasting when cholesterol was over 220 mg/dl or when the patient was currently being treated with hypolipidemic agents.

The control group comprised healthy volunteers with no stroke or history of stroke, or any etiology of optic neuritis, and a normal computed-tomography brain scan. A standard neurologic, ophthalmological, and fundoscopic examination documented the absence of stroke or neuritis, and a standardized questionnaire assessed the absence of previous ischemic cardio-cerebro-vascular events. They were matched to the cases for age and gender, and were evaluated for hypertension, diabetes mellitus, and dyslipidemia.

All patients and controls were free from autoimmune diseases and negative for lupus anticoagulant and other coagulopathies.

The study protocol was in accordance to the Helsinki declaration, approved by the Institutional Protocol and Ethics Review Committee. All participants gave informed consent.

### Genetic analysis

Whole blood samples from both patients and controls were used for isolation of peripheral blood leukocytes for genetic analysis. Genomic DNA (hereafter DNA) from each individual was isolated using a standard NaCl extraction procedure. Briefly, in a 1.5 ml tube with 700 μl of peripheral blood, 700 μl of lysis buffer (10 mM Tris-HCl pH 7.8, 2 mM EDTA (Invitrogen, Carlsbad, CA) 10 mM KCl, 4 mM MgCl_2_ [Merk Chemical, Darmstadt, Germany]) and 2 drops of IGEPAL CA-630 (Sigma-Aldrich Company LTD, UK) were added, were gently vortexed and centrifuged at 6500× g for 1 min. The supernatant was removed and the pellet resuspended in 500 μl of lysis buffer and centrifuged at 6500× g for 1 s (wash step). The wash step was repeated for 3–5 times. After the washes the pellet was resuspended in 200 μl of lysis buffer and 15 μl 15% SDS (Sigma-Aldrich Company LTD) and was placed in waterbath at 55 °C for 5 min.

Following incubation 85 μl of NaCl 6M (Merk Chemical) were added and centrifuged at 9600× g for 3 min. The supernatant was resuspended at 750 μl of ice-cold 100% ethanol (Sigma-Aldrich Company LTD) to precipitate the DNA. The precipitated DNA was transferred to another 1.5 ml tube with 50 μl of TE (10 mM Tris-HCl pH 7.8, 1 mM EDTA pH 8.0-Invitrogen).

The I/D polymorphism in intron 16 of the *ACE* gene was analyzed according to Rigat [19]. To determine the *ACE* insertion/deletion genotype, PCR amplification was performed in a total volume of 25 μl consisting of 150 ng extracted DNA, 0.2 mM Deoxynucleotide Triphosphates (dNTPs; Invitrogen), 10 pmol of each primer (Invitrogen), 10× Taq DNA polymerase buffer (Invitrogen), 2 mM MgCl_2_ (Invitrogen), 5% dimethyl sulfoxide DMSO (Euroclone, Milano, Italy), and 1.25 units of Taq DNA polymerase (Invitrogen). The forward and reverse primers were, respectively: 5′-CTG GAG ACC ACT CCC ATC CTT CTC-3′ and 5′-GAT GTG GCC ATC ACA TTC GTC AGA T-3′. The thermal cycling was as follows: 10 min denaturation at 94 °C, 30 cycles of 94 °C for 45 s, 58 °C for 30 s, and 72 °C for 45 s, with a final extension at 72 °C for 10 min. The final products, one of 490 bp with the insert (I allele) and one of 190 bp without the insert (D allele), were separated on 2% agarose gel. The resulting genotypes were DD, ID, and II.

For *AGT* M235T allele determination, PCR was performed according to a previously described procedure [20]. Briefly, the *AGT* M235T genotype was determined by PCR amplification in a 25 μl mixture of 150 ng of extracted DNA, 0.2 mM dNTPs (Invitrogen), 5 pmol of each primer (forward: 5′-CAG GGT GCT GTC CAC ACT GGA CCC C-3′; reverse: 5′-CCG TTT GTG CAG GGC CTG GCT CTC T-3′), 10x Taq DNA polymerase buffer (Invitrogen), 1.5 mM MgCl_2_ (Invitrogen), 5% DMSO (Euroclone), and 1 unit of Taq DNA polymerase (Invitrogen). The thermocycling conditions were as follows: at 90 °C for 3 min, 10 cycles at 94 °C for 1 min, at 68 °C for 1 min, and at 72 °C for 1 min, then continued with 30 cycles at 90 °C for 30 s, at 68 °C for 1 min, and at 72 °C for 30 s, with a final extension at 72 °C for 10 min. The PCR products were digested at 37 °C for 6 h with the restriction enzyme Tth111I (New England Biolabs-NEB, Hitchin, UK). Digestion products were separated by 2% agarose gel electrophoresis and visualized by exposure to ultraviolet light after ethidiun bromide (Invitrogen) staining. The lengths of the separated fragments were 165 bp, corresponding to the M235 allele (M), and 141 bp, corresponding to the digested T235 allele (T). The resulting genotypes were MM, MT, and TT.

To determine AT1-receptor A1166C genotypes, PCR amplification was also performed using standard procedures [21]. PCR amplification was performed in a total volume of 25 μl consisting of 150 ng extracted DNA, 0.2 mM dNTPs (Invitrogen), 4 pmol of each primer (Invitrogen), 10x Taq DNA polymerase buffer (Invitrogen), 2 mM MgCl_2_ (Invitrogen), and one unit of Taq DNA polymerase (Invitrogen). The forward and reverse primers were: 5′-GCA CCA TGT TTT GAG GTT-3′ and 5′-CGA CTA CTG CTT AGC ATA-3′, respectively. The reaction was performed using the following protocol: denaturation for 5 min at 94 °C, followed by 40 cycles of denaturation (94 °C, 30 s), annealing (52 °C, 30 s), and extension (72 °C, 30 s), and a final 10 min extension at 72 °C. The amplified product was digested for 3 h at 37 °C with the Dde1 (New England Biolabs-NEB) restriction enzyme, electrophoresed in 2% agarose gel, and visualized by staining with ethidium bromide (Invitrogen). Amplification of the A allele resulted in a 546 bp DNA fragment.

Digestion of the C allele resulted in a 435 bp DNA fragment. The resulting genotypes were AA, AC, and CC. All samples were run in duplicates with positive and negative for each genotype sample as controls and blanks.

### Statistical analysis

The gene counting method estimated allele frequencies at the individual loci. The agreement of genotype frequencies with Hardy–Weinberg equilibrium expectations was tested using the χ^2^ test. Statistical analysis was performed using the statXact program. All p values were two-tailed and were considered statistically significant at p<0.05. Confidence intervals (CIs) were calculated at the 95% level. Odds ratios (ORs) were defined as the odds ratio of patients with NAION divided by the odds of the control groups, and were calculated to provide an estimate of the relative risk of *RAAS* polymorphisms associated with NAION.

## Results

Forty-seven patients (29 men and 18 women) and 76 controls (47 men and 29 women) were recruited for the study. The patients’ ages ranged from 49 to 85 years, with a mean age of 66.2 years. The age range of the controls was from 48 to 75 years, with a mean age of 65.6 years. Most cases were older than 55 years, and only eight patients were younger (six men and two women). In the control group, 11 individuals (eight men and three women) were under 55 years of age. Epidemiological and other relevant clinical data are shown in Table 1.

**Table 1:** Clinical characteristics of patients and controls

**Data**	**Cases (n=47)**	**Controls (n=76)**
Males	29 (61.7)	47 (61.8)
Females	18 (38.3)	29 (38.1)
Age, mean	66.2	65.6
Hypertension	28 (59.5)	42 (55.2)
Diabetes mellitus	12 (25.5)	19 (25)
Dyslipidemia	20 (42.5)	31 (40.7)

Numbers in parentheses for nominal data indicate percentages and for continuous ±SD

The polymorphisms were in Hardy–Weinberg equilibrium in the study population and the control group. The genotype and allele distributions are shown in Table 2. No statistically significant difference, regarding genotypes and alleles, was found for the three polymorphisms between the control and patient groups.

**Table 2:** Genotype and allele distributions of RAS polymorphisms in patients and controls

**Genotypes/alleles**	**Patients (47)**	**Controls (76)**
ID	26 (55.3%)	34 (44.7%)
DD	14 (29.8%)	30 (39.5%)
II	7 (14.9%)	12 (15.8%)
AC	20 (42.6%)	31 (40.8%)
AA	25 (53.2%)	40 (52.6%)
CC	2 (4.2%)	5 (6.6%)
MT	23 (48.9%)	40 (52.6%)
MM	17 (36.2%)	24 (31.6%)
TT	7 (14.9%)	12 (15.8%)
D allele presence	40 (85.1%)	64 (84.2%)
I allele presence	33 (70.2%)	46 (60.5%)
A allele presence	45 (95.7%)	71 (93.4%)
C allele presence	22 (46.8%)	36 (47.3%)
M allele presence	40 (85.1%)	64 (84.2%)
T allele presence	30 (63.8%)	52 (68.4%)

Despite these negative results, when correlations were made according to age and gender (Table 3), some noteworthy findings emerged for the I/D polymorphism.

**Table 3:** I/D genotypes and alleles in regard to gender and age

**Genotypes alleles**	**Cases** **(n=47)**	**Controls** **(n=76)**	**Cases ≤55 y (n=8)**	**Controls≤55 y** **(n=11)**	**Male cases (n=29)**	**Female cases** **(n=18)**	**Male controls** **(n=47)**	**Male cases ≤55 y (n=6)**
DD genotype	14 (29.8%)	30 (39.5%)	1/8 (12.5%)	3/11 (27.2%)	9/29 (31%)	5/18 (27.7%)	14/47 (29.7%)	0/6 (0%)
II genotype	**7 (14.9%)**	**12 (15.8%)**	**4/8 (5%)**	**2/11 (18.1%)**	**5/29 (17.2%)**	2/18 (11.2%)	**6/47 (12.7%)**	**4/6 (66.7%)**
D allele presence	40 (85.1%)	64 (84.2%)	4/8 (5%)	9/11 (81.8%)	24/29 (82.75%)	16/18 (88.8%)	41 (87.2%)	2/6 (33.3%)
I allele presence	33 (70.2%)	46 (60.5%)	7/8 (87.5%)	20/29 (72.7%)	20/29 (68.9%)	13/18 (72.2%)	33 (70.2%)	**6/6 (100%)**

Regarding age, the II genotype frequency was higher in the younger age group ≤50 years (50%), compared to the cases (14.9%), the whole controls (15.8%), and the subgroup of controls ≤55 years (18.1%). This difference was statistically significant when patients at or below 55 years were compared to all cases and the controls, while there was no significant difference in comparison to the controls at or below 55 years (p=0.033, OR=5.71, CI=1.152–28.35; p=0.03, OR=5.33, CI: 1.170–24.31 and p=0.15, respectively).

Regarding gender, no significant difference was found when male cases were compared to male controls. When comparisons were made according to age and gender simultaneously, there was a significant difference in the frequency of the II genotype in male cases at or below 55 years (66.6%) in comparison to all male cases and the male controls (p=0.023, OR=9.6, CI: 1.363–67.60 and p=0.007, OR=13.67, CI: 2.04–91.49, respectively).

Allele I of the *ACE* gene was more frequently present in patients, compared to controls (72.3% in cases versus 60.5% in controls), especially in younger patients under 55 years of age (87.5% in patients at ≤55 years versus 72.7% in controls at ≤55 years), but without statistical significance. Following an age-gender approach, comparison of male patients under 55 years to all male cases showed that the I allele was present in all six younger male patients (100%).

Regarding the M235T polymorphism of the *AGT* gene and the A1166C the polymorphism of the *AT1-receptor* gene, any correlation between gender and age groups disclosed no statistically significant difference in allele and genotype distributions. As a result, no association of these two polymorphisms with NAION occurrence was identified.

## Discussion

A finding that surfaced from our study concerned the *ACE* I/D polymorphism, suggesting that the II genotype is a risk factor for NAION in younger patients, especially men, and that the I allele is likely a predisposing factor for NAION in younger males, but not in younger females (≤55 years). We did not find any association between the M235T *AGT* polymorphism, the A1166C *AT1-receptor* polymorphism, and NAION occurrence

Although apoplectic onset, disc swelling, hyperemia, and splinter hemorrhages support infarction as the precipitating event in NAION, the precise mechanism of optic nerve ischemia has not been absolutely clarified, and the contribution of the various known vascular risk factors is not entirely known. The optic nerve disc has a unique vasculature with contributions from the short posterior ciliary arteries, the pial circulation, and the retinal circulation [22], with an autoregulatory mechanism controlling blood flow. This arrangement produces watershed zones at the boundaries of supply from adjacent circulations. When this mechanism is deficient, ischemia may occur. It has been suggested that NAION may be the result of a compromised watershed microcirculation, via mechanisms different from cerebrovascular and cardiovascular events. NAION is not clearly a thrombotic or an embolic disease, as has been shown by studies demonstrating no increased risk of cerebrovascular disease in NAION patients [3,23].

Recent literature indicates that NAION is associated with nocturnal arterial hypotension, which may produce hypoperfusion of the optic nerve head microvasculature [24]. NAION development within hours of using phosphodiesterase type-5 inhibitors has also been described [25], with the blunting of vasculoprotective actions of nitric oxide through cyclic-GMP hydrolysis by the phosphodiesterase type-5 inhibitor being a possible mechanism [26]. Therefore, altered vascular regulation, due to nitric oxideconcentration changes, may result in NAION in particular patients. Consequently, insufficient or altered autoregulatory circulation mechanisms may provoke NAION development. Polymorphisms of genes involved in systematic circulation, such as the *RAAS* and vasculoprotective genes, may, like the *NOS* genes, have a role in NAION occurrence, although results conflict [27,28].

The rennin-angiotensin-aldosterone system plays a central role in blood pressure regulation, and *RAAS* genes have been shown to affect blood pressure phenotypes [29,30]. The *ACE* I/D polymorphism appears to influence *ACE* expression and blood pressure, with the D allele and DD genotype being associated with high blood pressure, while the II genotype and the I allele are associated with lower blood pressure [31,32]. The T allele of the M235T polymorphism of *AGT* is also associated with increased blood pressure [33,34], whereas contradictory evidence exists for the A1166C polymorphism of the *AT1-receptor* gene [35,36]. Furthermore, previous studies have demonstrated that the RAAS genotypes have a gender dimorphism concerning their influence on ACE plasma concentration [37], the molar ratio of Ang II to Ang-(1–7) [38], and the renin and pro-renin concentrations [39], suggesting that gene regulation of RAAS is affected by the gonadal steroids [40,41]. As a result, in many studies, the DD genotype is associated with an increased risk of cardiac hypertrophy and hypertension among men, without a similar association being present among women [41,42].

Our results do not indicate that certain *RAAS* polymorphisms play a role in NAION onset. The suspected influence on the I allele and II genotype in ages below 55 years is intriguing and may support the hypothesis that hypoperfusion and low pressure are possible underlying causes of NAION in this age group [30]. A possible unfavorable effect of the II genotype in males is also present, in accordance with the aforementioned sexual dimorphism of the *RAAS* genes [41,42].

The small number of patients younger than 55 years made the comparisons less conclusive, and the small number of younger women did not permit any comparisons between younger and older women or between younger men and women. The strength of our study is its use of an homogenous population affected only with limited immigration the last century.

In summary, our study, despite some negative results and other limitations, uncovered interesting findings on blood-pressure gene polymorphisms and NAION susceptibility. It is, to our knowledge, the first study to attempt gender- and age-related analysis of these patients. Large-scale, multicentered, controlled prospective studies are needed to further explore the effects of *RAAS* polymorphisms and other genetic factors on NAION susceptibility.
